# Social orienting is reduced in williams syndrome

**DOI:** 10.1192/j.eurpsy.2021.350

**Published:** 2021-08-13

**Authors:** J. Kleberg, D. Riby, A. Norgren

**Affiliations:** 1 Department Of Clinical Neuroscience, Karolinska institutet, Stockholm, Sweden; 2 Department Of Molecular Medicine And Surgery, Karolinska institutet, Stockholm, Sweden; 3 Centre For Developmental Disorders Department Of Psychology, Durham University, Durham, United Kingdom

**Keywords:** visual attention, Williams syndrome, Rare genetic syndromes, face processing

## Abstract

**Introduction:**

Williams syndrome (WS) is a rare genetic disorder caused by a deletion at chromosome 7q1123. WS is associated with high empathy, relatively good face memory and low social anxiety. Despite these strengths, WS individuals typically have an intellectual disability, difficulties with visuospatial perception, non-social anxiety and complex social cognition. Attention to other’s eyes is crucial for adaptive social understanding. Consequently, eyes trigger quick and automatic gaze shifts in typically developing individuals. It is not known whether this process is atypical in WS.

**Objectives:**

To examine visual attention to other’s eyes in Williams syndrome.

**Methods:**

Individuals with WS (n = 35; mean age 23.5 years) were compared to controls (n = 167) in stratified age groups (7 month, 8-12 years, 13-17 years, adults). Participants were primed to look at either the eyes or the mouth of human faces. The latency and likelihood of a first gaze shift from, or to the eyes, was measured with eye tracking.

**Results:**

WS individuals were less likely, and slower to orient to the eyes than typically developing controls in all age groups from eight years of age (all p <.001), but did not differ from 7 months old infants. In contrast to healthy individuals from eight years and above, WS individuals did not show a preference to orient towards the eyes relative to the mouth.

**Conclusions:**

Despite the hyper-social behavioral phenotype, WS is associated with reduced attention to other’s eyes during early stages of processing. This could contribute to the difficulties with complex social cognition observed in this group.

**Disclosure:**

No significant relationships.

